# Bridging the Technological Divide in Medicine: A Global Responsibility

**DOI:** 10.1055/s-0044-1795168

**Published:** 2024-12-20

**Authors:** Dale Dangleben

**Affiliations:** 1 Surgical Critical Care, Penn State Health, Camp Hill, Pennsylvania

**Figure:**
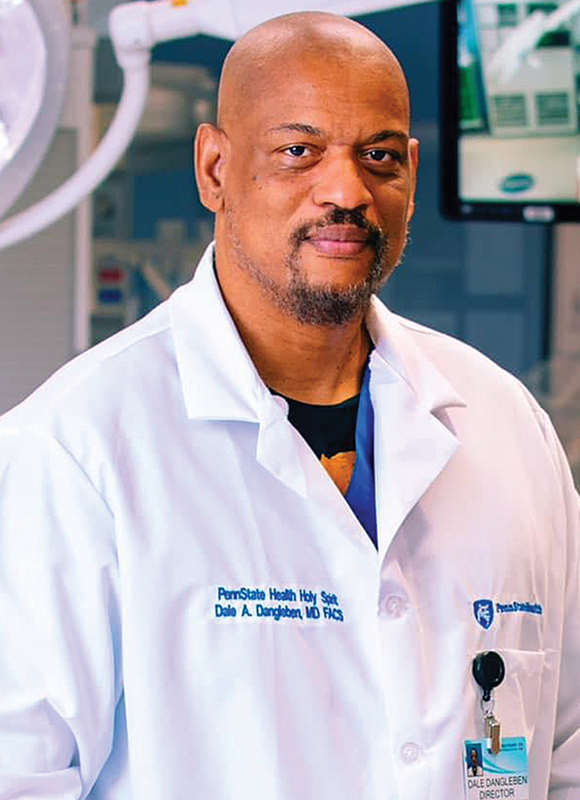
Dale Dangleben, MD, FACS

In an era where technology is revolutionizing medicine at an unprecedented pace, the gap between developed and less developed countries in accessing and implementing these advancements is becoming increasingly stark. Innovations like artificial intelligence (AI), telesurgery, and other cutting-edge medical technologies hold the potential to transform health care by making it more efficient, precise, and accessible. However, without intentional efforts to ensure these benefits reach all corners of the globe, we risk widening existing disparities in health care outcomes.

The question, therefore, is not just how we continue to push the boundaries of what is possible in medicine, but how we bring the world along with us, ensuring that no nation or population is left behind. Developed countries have a moral obligation, as well as a practical interest, in assisting less developed countries to catch up with these technological advances. Here are some ways this can be achieved.

## Collaborative Partnerships

One of the most effective strategies is fostering partnerships between institutions in developed and less developed countries. These partnerships can take many forms, from academic exchanges to joint research projects and technology transfer agreements. Developed countries, with their vast resources and expertise, can help build the technological infrastructure necessary in less developed regions. This includes providing not only the hardware and software required for technologies like AI and telesurgery but also the training and support systems needed to use these tools effectively.

Such collaborations should be designed with sustainability in mind, empowering local health care providers to become self-sufficient over time. This approach not only builds local capacity but also ensures that the solutions implemented are tailored to the specific needs and contexts of the regions they are intended to serve.

## Education and Training

Access to technology is only one part of the equation; the ability to use and apply these technologies is equally crucial. Developed countries can play a pivotal role by offering educational and training programs focused on the latest advancements in medical technology. This could include online courses, webinars, and remote mentoring, leveraging the same technologies they aim to disseminate, such as telesurgery platforms and AI-driven learning tools.

By investing in the education of health care professionals in less developed countries, we not only enhance their ability to use current technologies but also prepare them to contribute to future innovations. It is important that these educational initiatives are accessible and culturally appropriate, ensuring they meet the needs of diverse populations.

## Affordability and Accessibility

Cost is a significant barrier to the adoption of advanced medical technologies in less developed countries. Many of these nations struggle with limited health care budgets, making it difficult to justify the purchase of expensive equipment or the implementation of sophisticated systems like AI. Developed countries and international organizations can help by providing subsidies, grants, or low-interest loans specifically for the acquisition of medical technologies.

Moreover, technology companies should be encouraged to develop and offer more affordable versions of their products for markets in less developed countries. This could involve creating scalable solutions that are less expensive to produce and maintain, or developing open-source technologies that can be freely accessed and modified to suit local needs.

## Policy and Advocacy

Governments in developed countries can support these efforts by prioritizing policies that promote global health equity. This includes advocating for international agreements that facilitate the sharing of medical technologies and expertise, as well as providing incentives for companies and institutions to engage in these efforts.

Additionally, developed countries should work with global health organizations to ensure that technological advancements are integrated into existing global health initiatives. For example, AI could be used to enhance disease surveillance systems in less developed countries, improving their ability to respond to outbreaks and manage public health challenges.

## Innovation Hubs and Local Empowerment

While it is crucial for developed countries to assist, it is equally important to empower less developed countries to drive their own innovations. Supporting the establishment of local innovation hubs, where technology and health care intersect, can foster homegrown solutions that are more attuned to the specific challenges and opportunities of the region.

These hubs could be funded by a combination of international aid, private investment, and local governments, creating a sustainable model for ongoing technological development. Encouraging entrepreneurship in the medical technology sector within these regions can also lead to the creation of new tools and systems that could benefit not only the local population but the world at large.

## Conclusion

The rapid advancement of technology in medicine offers an extraordinary opportunity to improve health care worldwide. However, it also presents a significant challenge: ensuring that these advancements benefit all people, not just those in developed countries. By focusing on collaborative partnerships, education, affordability, policy advocacy, and local empowerment, developed countries can help bridge the technological divide, creating a more equitable global health care landscape.

In the interconnected world we live in, no country exists in isolation. The health of one nation inevitably impacts the health of others, making it imperative that we work together to ensure that the benefits of medical technology are shared universally. Only by doing so can we truly realize the full potential of these advancements and build a healthier future for everyone.

